# Distribution of *CYP2C19* genetic polymorphisms and pharmacogenomic implications in 11,710 patients with cardiovascular and cerebrovascular conditions: a large population-based study in eastern China

**DOI:** 10.3389/fgene.2026.1830873

**Published:** 2026-06-29

**Authors:** Minfei Peng, Minmin He, Ying Chen, Xiaoli Zhu, Jun Li

**Affiliations:** 1 Department of Laboratory Medicine, Taizhou Hospital of Zhejiang Province Affiliated to Wenzhou Medical University, Linhai, China; 2 Key Laboratory of System Medicine and Precision Diagnosis and Treatment of Taizhou, Taizhou, China

**Keywords:** clopidogrel, *CYP2C19*, genetic epidemiology, genetic polymorphism, pharmacogenomics

## Abstract

**Objective:**

To investigate the distribution of *CYP2C19* genetic polymorphisms among patients with cardiovascular and cerebrovascular conditions in Taizhou, Zhejiang Province, and to evaluate their pharmacogenomic implications for genotype-guided antiplatelet therapy.

**Methods:**

We conducted a retrospective population-based analysis of 11,710 patients with cardiovascular and cerebrovascular conditions who underwent *CYP2C19* genotyping at Taizhou Hospital of Zhejiang Province between January 2023 and December 2025. Genotyping of CYP2C19*2 and CYP2C19*3 alleles was performed using fluorescence-based quantitative polymerase chain reaction (PCR). Genotype and allele frequencies were calculated using the gene counting method. Hardy–Weinberg equilibrium (HWE) was assessed using the χ^2^ test. Differences in genotype and phenotype distributions across gender and age groups were analyzed using the χ^2^ test, and P < 0.05 was considered statistically significant.

**Results:**

The allele frequencies of CYP2C19*1, *2, and *3 were 61.01%, 34.11%, and 4.88%, respectively. The distribution of metabolic phenotypes was as follows: normal metabolizers (NMs), 37.15%; intermediate metabolizers (IMs), 47.73%; and poor metabolizers (PMs), 15.12%. Genotype distributions were consistent with Hardy–Weinberg equilibrium (P > 0.05). No significant differences were observed in genotype or phenotype distributions across gender or age groups (P > 0.05). Regional comparisons showed that the distribution pattern in Taizhou was consistent with that reported in other regions of eastern China but differed from southern and western populations (P < 0.05). The proportion of individuals carrying at least one loss-of-function allele (IMs + PMs) reached 62.85%, indicating a high prevalence of genotypes associated with reduced clopidogrel responsiveness.

**Conclusion:**

This large population-based pharmacogenomic study demonstrates a high prevalence of *CYP2C19* loss-of-function alleles in patients with cardiovascular and cerebrovascular conditions from Taizhou. These findings support the clinical value of *CYP2C19* genotyping for individualized antiplatelet therapy and provide important genetic epidemiology data for precision medicine in this population.

## Introduction

Cardiovascular disease (CVD) remains the leading cause of morbidity and mortality worldwide and represents a substantial public health burden in China. In recent years, both the prevalence and the incidence of CVD in China have continued to rise, with an observable trend toward younger onset (Zhou et al., 2019). Percutaneous coronary intervention (PCI) has become a cornerstone therapy for acute coronary syndrome (ACS) and stable coronary artery disease; following PCI, dual antiplatelet therapy (DAPT), typically consisting of aspirin and a P2Y12 receptor inhibitor, is essential for preventing stent thrombosis and major adverse cardiovascular events (MACE) (Siasos et al., 2015).

Precision medicine aims to optimize disease prevention and treatment by considering individual variability in genetic, environmental, and lifestyle factors. Pharmacogenomics, as an important component of precision medicine, focuses on how genetic variation influences drug response and thereby provides a basis for genotype-guided therapy, particularly in cardiovascular medicine (Leopold and Loscalzo, 2018; Naithani et al., 2021).

Clopidogrel, a second-generation thienopyridine prodrug, remains widely used as a P2Y12 inhibitor because of its established efficacy, safety profile, and cost-effectiveness. However, substantial inter-individual variability in clopidogrel responsiveness has been consistently reported. Approximately 20%–40% of patients exhibit high on-treatment platelet reactivity, which is associated with an increased risk of thrombotic complications after PCI. This variability is largely attributable to genetic polymorphisms affecting clopidogrel bioactivation.

Clopidogrel requires hepatic biotransformation through a two-step oxidative process mediated by cytochrome P450 (CYP) enzymes to generate its active metabolite. Among these enzymes, *CYP2C19* plays a pivotal role in determining the plasma concentration of the active metabolite and the degree of platelet inhibition (Castrichini et al., 2023). Recent guidelines have increasingly highlighted the importance of individualized antiplatelet strategies in cerebrovascular disease. In particular, the 2026 American Heart Association/American Stroke Association Guideline for the Early Management of Patients With Acute Ischemic Stroke emphasizes early secondary prevention as an essential component of acute stroke care, underscoring the potential value of genotype-guided antiplatelet therapy (Prabhakaran et al., 2026). The *CYP2C19* gene is highly polymorphic, and several variants have been shown to significantly alter enzymatic activity.

The most clinically relevant loss-of-function (LOF) alleles are CYP2C19*2 and CYP2C19*3, which result in splicing defects or premature termination codons, leading to reduced or absent enzymatic function (Pan et al., 2017; Botton et al., 2021). According to the Clinical Pharmacogenetics Implementation Consortium (CPIC) guidelines, individuals are classified as normal metabolizers (NMs), intermediate metabolizers (IMs), or poor metabolizers (PMs) based on genotype. Carriers of one or two LOF alleles (IMs and PMs) exhibit reduced generation of the active metabolite and diminished platelet inhibition, which are associated with increased risks of stent thrombosis and recurrent ischemic events (Voora and Ginsburg, 2012; Xi et al., 2019). Regulatory agencies and international guidelines increasingly recommend genotype-guided antiplatelet therapy in appropriate clinical settings.

Importantly, the distribution of *CYP2C19* polymorphisms varies substantially across ethnicities and geographic regions (Dorji et al., 2019). East Asian populations have a markedly higher prevalence of LOF alleles compared with European and African populations. This genetic background contributes to differences in drug response and may partially explain the so-called “East Asian paradox,” characterized by distinct thrombotic and bleeding risk profiles (Kwon and Park, 2022).

Although several regional studies have reported *CYP2C19* genotype distributions in different parts of China, large-scale pharmacogenomic data from Taizhou, Zhejiang Province, remain scarce. Given the potential regional genetic heterogeneity within China, locally derived data are essential for optimizing precision antiplatelet strategies.

Therefore, the present study aimed to investigate the distribution of *CYP2C19* genetic polymorphisms in a large cohort of patients with cardiovascular and cerebrovascular conditions in Taizhou, Zhejiang, and to explore their potential clinical relevance for genotype-guided antiplatelet therapy.

## Materials and methods

### Study population

A total of 11,710 patients with cardiovascular and cerebrovascular conditions who underwent *CYP2C19* genetic testing at Taizhou Hospital of Zhejiang Province between January 2023 and December 2025 were enrolled in this study. Based on the main clinical indication for testing, the study population was broadly categorized into coronary artery disease/acute coronary syndrome/percutaneous coronary intervention-related conditions (35.39%), peripheral arterial disease (29.36%), ischemic stroke/transient ischemic attack (16.09%), and other cerebrovascular diseases (19.16%). The cohort included 7,245 males (61.87%) and 4,465 females (38.13%), with a mean age of 65.57 ± 11.53 years. All participants were unrelated and reported no family history of hereditary diseases. The inclusion criteria were: (1) age ≥ 18 years; (2) having cardiovascular or cerebrovascular conditions requiring clinical evaluation for antiplatelet therapy; and (3) having a clinical indication for antiplatelet therapy and *CYP2C19* testing. Exclusion criteria included: (1) related family members; (2) missing clinical data; or (3) uninterpretable genetic testing results.

### Genotyping procedures

Exfoliated buccal mucosal cells were collected from the patients. Sampling was performed using disposable sterile swabs to scrape the bilateral buccal mucosa, which were then immediately placed into tubes containing lysis buffer.

Genotyping of CYP2C19*2 (c.681G>A) and CYP2C19*3 (c.636G>A) alleles was performed using a fluorescence-based quantitative polymerase chain reaction (PCR) assay with a commercially available *CYP2C19* Gene Detection Kit (Chongqing King-Yin Biotechnology Co., Ltd., Chongqing, China). Amplification reactions were conducted on the JY-1000 A automated medical PCR analysis system according to the manufacturer’s instructions.

Each run included positive controls with known genotypes and negative controls to ensure assay accuracy. Genotypes were automatically interpreted using integrated analysis software.

### Phenotype classification

Metabolic phenotypes were inferred according to CPIC guidelines based on CYP2C19*2 and *3 genotype combinations. Normal metabolizers (NMs) were defined as individuals with the *1/*1 genotype, intermediate metabolizers (IMs) as those with the *1/*2 or *1/*3 genotype, and poor metabolizers (PMs) as those with the *2/*2, *2/*3, or *3/*3 genotype.

The terminology “normal metabolizer” was adopted in accordance with current international consensus nomenclature, replacing the previously used term “extensive metabolizer.”

### Statistical analysis

Statistical analyses were performed using SPSS version 26.0 (IBM Corp., Armonk, NY, United States).

Genotype and allele frequencies were calculated using the gene counting method. Hardy–Weinberg equilibrium (HWE) was assessed using the χ^2^ test.

Categorical variables are presented as numbers and percentages [n (%)]. Differences in genotype and phenotype distributions across gender and age groups were evaluated using the χ^2^ test. A two-sided P value < 0.05 was considered statistically significant.

### Ethics statement

This study was approved by the Medical Ethics Committee of Taizhou Hospital of Zhejiang Province (Approval No. K20260129).

## Results

### Genotype distribution and Hardy–Weinberg equilibrium

An analysis of the 11,710 patient samples from the Taizhou area revealed that the observed genotype distributions for CYP2C19*2 (*P* = 0.18) and CYP2C19*3 (*P* = 0.86) did not significantly deviate from the expected values (*P* > 0.05). These results indicate that the population is in Hardy–Weinberg equilibrium (HWE), suggesting that the study cohort was genetically representative of the analyzed population (Table 1).

**TABLE 1:** Hardy–Weinberg equilibrium analysis of CYP2C19*2 and CYP2C19*3 alleles in the Taizhou population.

Gene locus	Genotype	Observed (n)	Expected (n)	Frequency (%)	*P-value*
CYP2C19*2	GG	5,060	5,082	43.21	0.18
	GA	5,311	5,260	45.35	
	AA	1,339	1,368	11.43	
CYP2C19*3	GG	10598	10599	90.50	0.86
	GA	1,082	1,086	9.24	
	AA	30	26	0.26	

Abbreviations: HWE, Hardy–Weinberg equilibrium.

### Influence of gender and age on genotype distribution

A total of six genotypes were identified in the study population. The single heterozygous mutation genotype, *1/*2, showed the highest frequency at 41.92% (n = 4,909), followed by the wild-type homozygous genotype *1/*1 at 37.15% (n = 4,350). The distribution of homozygous and compound mutant genotypes was as follows: *2/*2 (11.43%), *1/*3 (5.81%), *2/*3 (3.43%), and *3/*3, which had the lowest prevalence at 0.26%. Regarding allele frequencies, CYP2C19*1 was the most prevalent (61.01%), followed by the LOF alleles *2 (34.11%) and *3 (4.88%).

No significant differences were observed in genotype distributions between male and female patients (χ^2^ = 1.788, *P* = 0.878). Similarly, allele frequencies did not differ significantly by gender (χ^2^ = 1.592, *P* = 0.451). When stratified into four age groups (≤40, 41–60, 61–80, and >80 years), genotype distributions showed no statistically significant variation (χ^2^ = 15.089, *P* = 0.445). Allele frequencies were likewise comparable across all age cohorts (χ^2^ = 2.927, *P* = 0.818). These findings indicate that the distribution of *CYP2C19* genetic polymorphisms in the Taizhou population is independent of gender and age (Table 2).

**TABLE 2:** Distribution of *CYP2C19* genotypes and allele frequencies stratified by gender and age.

Characteristics	*n*	Genotype [*n* (%)]						Allele [*n* (%)]		
		*1/*1	*1/*2	*1/*3	*2/*2	*2/*3	*3/*3	*1	*2	*3
Gender										
Male	7,245	2,699	3,031	430	813	252	20	8,859	4,909	722
		(37.25)	(41.84)	(5.94)	(11.22)	(3.48)	(0.28)	(61.14)	(33.88)	(4.98)
Female	4,465	1,651	1878	250	526	150	10	5,430	3,080	420
		(36.98)	(42.06)	(5.60)	(11.78)	(3.36)	(0.22)	(60.81)	(34.49)	(4.70)
*χ* ^*2*^	1.788							1.592		
*P-value*	0.878							0.451		
Age (years)										
≤40	268	106	110	11	29	12	0	333	180	23
		(39.55)	(41.04)	(4.10)	(10.82)	(4.48)	(0.00)	(62.13)	(33.58)	(4.29)
41–60	3,189	1,221	1,307	174	354	121	12	3,923	2,136	319
		(38.29)	(40.98)	(5.46)	(11.10)	(3.79)	(0.38)	(61.51)	(33.49)	(5.00)
61–80	7,047	2,596	2,972	412	819	233	15	8,576	4,843	675
		(36.84)	(42.17)	(5.85)	(11.62)	(3.31)	(0.21)	(60.85)	(34.36)	(4.79)
>80	1,206	427	520	83	137	36	3	1,457	830	125
		(35.41)	(43.12)	(6.88)	(11.36)	(2.98)	(0.25)	(60.41)	(34.41)	(5.18)
*χ* ^*2*^	15.089							2.927		
*P-value*	0.445							0.818		
Total [*n* (%)]	11710	4,350	4,909	680	1,339	402	30	14289	7,989	1,142
		(37.15)	(41.92)	(5.81)	(11.43)	(3.43)	(0.26)	(61.01)	(34.11)	(4.88)

### The distribution of *CYP2C19* phenotypes in patients with cardiovascular and cerebrovascular conditions

The distribution of genotype-inferred *CYP2C19* phenotypes showed that NMs (*1/*1), IMs (*1/*2 and *1/*3), and PMs (*2/*2, *2/*3, and *3/*3) accounted for 37.15%, 47.73%, and 15.12% of the study population, respectively. Among the six observed genotypes, *1/*2 was the most frequent (41.92%), followed by *1/*1 (37.15%), *2/*2 (11.43%), *1/*3 (5.81%), *2/*3 (3.43%), and *3/*3 (0.26%). Overall, individuals carrying at least one LOF allele (IMs and PMs) comprised 62.85% of the study population. No statistically significant differences in *CYP2C19* phenotype distribution were observed across gender (χ^2^ = 0.339, *P* = 0.844) or age groups (χ^2^ = 5.887, *P* = 0.436) (Table 3).

**TABLE 3:** Distribution of *CYP2C19* metabolic phenotypes stratified by gender and age.

Characteristics	*n*	NMs [*n* (%)]	IMs [n (%)]	PMs [*n* (%)]	*χ* ^*2*^	*P-value*
Overall	11,710	4,350 (37.15)	5,589 (47.73)	1,771 (15.12)	​	​
Gender						
Male	7,245	2,699 (37.25)	3,461 (47.77)	1,085 (14.98)	0.339	0.844
Female	4,465	1,651 (36.98)	2,128 (47.66)	686 (15.36)		
Age (years)						
≤40	268	106 (39.55)	121 (45.15)	41 (15.30)	5.887	0.436
41–60	3,189	1,221 (38.29)	1,481 (46.44)	487 (15.27)		
61–80	7,047	2,596 (36.84)	3,384 (48.02)	1,067 (15.14)		
>80	1,206	427 (35.41)	603 (50.00)	176 (14.59)		

Abbreviations: NMs, normal metabolizers; IMs, intermediate metabolizers; PMs, poor metabolizers.

### Regional and international comparisons

Comparison of our findings with data from other domestic regions showed that the distribution of *CYP2C19* metabolic phenotypes in Taizhou was consistent with those reported in Hubei Province (*P* = 0.124), Hebei Province (*P* = 0.439), and other areas within Zhejiang Province (*P* = 0.179). However, significant statistical differences were observed when compared to Guangdong Province (including Guangzhou, Dongguan, and Foshan), Shaanxi Province, and Xinjiang Province (including the Kashgar region) (all *P* < 0.05), highlighting distinct regional distribution characteristics (Table 4).

**TABLE 4:** Comparison of *CYP2C19* metabolic phenotype distributions between the Taizhou population and other regions in China.

Region/Study	*n*	Metabolic phenotype distribution			*χ* ^*2*^	*P-value*	References
		NMs(n)	IMs(n)	PMs(n)			
Present study	11,710	4,350	5,589	1,771	​	​	This study
Hubei	1,036	418	468	150	4.175	0.124	Huan and Wang (2021)
Guangzhou	1783	710	845	228	8.686	0.013	Fang et al. (2017)
Dongguan	1,662	713	740	209	22.261	<0.001	Hua et al. (2017)
Guangdong	6,686	2,790	3,023	873	41.410	<0.001	Zhong et al. (2017)
Guangzhou	2,312	958	1,056	298	17.632	<0.001	Xiao et al. (2017)
Kashgar	1,020	591	361	68	181.523	<0.001	Li et al. (2014)
Shaanxi	4,248	1749	1996	503	37.148	<0.001	Ma et al. (2020)
Guangzhou	1899	765	878	256	8.006	0.018	Huang et al. (2023)
Xinjiang	1,068	454	514	100	29.697	<0.001	Xie et al. (2013)
Zhejiang	1,127	393	566	159	3.437	0.179	Hu et al. (2012)
Hebei	1,000	373	488	136	1.649	0.439	
Foshan	1,231	511	551	169	9.160	0.010	Yuan et al. (2022)

Abbreviations: NMs, normal metabolizers; IMs, intermediate metabolizers; PMs, poor metabolizers.

Globally, the frequency of PMs in the Taizhou population (15.12%) was slightly higher than the East Asian average (13%) and appeared to be higher than those reported in several non-East Asian populations, including Central/South Asia (8%), Africa (5%), and Europe (2%). This distribution pattern is consistent with the relatively high prevalence of *CYP2C19* loss-of-function alleles reported in East Asian populations.

## Discussion

In this large population-based pharmacogenomic study, we analyzed the distribution of *CYP2C19* polymorphisms in 11,710 patients with cardiovascular and cerebrovascular conditions and evaluated their potential impact on antiplatelet therapy. These findings provide important regional genetic epidemiology data to support individualized antiplatelet therapy.

### Principal findings

*CYP2C19* is a principal enzyme responsible for the oxidative activation of clopidogrel, and inter-individual variability in its activity is a major determinant of treatment response. To date, more than 39 *CYP2C19* allelic variants have been identified. Among these, *1 represents the wild-type allele with normal function, whereas the CYP2C19*2 and CYP2C19*3 alleles are associated with reduced bioactivation and diminished therapeutic efficacy (Zhuo et al., 2018). Therefore, characterizing the regional distribution of *CYP2C19* genetic polymorphisms is essential for informing precision antiplatelet therapy.

This retrospective analysis of *CYP2C19* genotypes in 11,710 patients with cardiovascular and cerebrovascular conditions in the Taizhou area represents the largest-scale study of *CYP2C19* genetic polymorphisms in this region to date. Our results show that the distributions of the CYP2C19*2 and *3 loci conform to Hardy-Weinberg equilibrium (*P* > 0.05), which suggests that the sample is representative of the regional population. The substantial sample size, wide age distribution, and balanced sex ratio enhance the generalizability of the findings to the regional population.

### Regional and ethnic differences

Studies have shown that the distribution of *CYP2C19* genetic polymorphisms exhibits ethnic and geographic variations (Gu et al., 2014). Comparative analyses revealed no significant differences in metabolic phenotype distribution between the Taizhou population and other research data from Hubei, Hebei, and other areas within Zhejiang province that are geographically adjacent or predominantly Han Chinese (*P* > 0.05). These findings suggest relative genetic homogeneity among Han Chinese populations in Central and Eastern China with respect to *CYP2C19* polymorphisms. However, significant statistical differences were observed compared to Guangdong (including Guangzhou, Dongguan, and Foshan) and Xinjiang (including Kashgar) (*P* < 0.05). These differences may reflect complex histories of population migration and evolution. Specifically, the Xinjiang region is closer to Central Asia, where populations with a higher frequency of normal metabolizer phenotypes have been reported. A study (Fricke-Galindo et al., 2016) also confirmed that the frequency of normal metabolizer genotypes is higher in Uyghur patients than in Han patients. The divergence between Guangdong and Taizhou may stem from genetic drift occurring within Southern Han populations over long-term evolutionary processes (Luan et al., 2026), although further evidence is required to confirm this hypothesis. These interregional and interethnic differences can also be interpreted from the perspective of pharmacoethnicity. Pharmacoethnicity refers to ethnic diversity in drug response or toxicity and is shaped by the combined influence of genetic background and non-genetic factors, including environmental exposures, local clinical practice patterns, and drug–drug interactions. In this context, the observed variability in *CYP2C19* polymorphisms across populations may contribute to differences in clopidogrel responsiveness and further supports the need for population-specific pharmacogenomic evidence in individualized antiplatelet therapy (O’Donnell and Dolan, 2009). China’s vast geographic territory, ethnic diversity, and complex demographic history likely contribute to the observed regional differences in *CYP2C19* polymorphism distribution.

In the global genetic landscape, research (Nieh and Roman, 2025) indicates that the frequency of CYP2C19*2 in Asian populations ranges from 20.5% to 53.8%, while CYP2C19*3 ranges from 0% to 15.6%. For example, Thai populations have been reported to show CYP2C19*2 and *3 allele frequencies of 25.36% and 4.50%, respectively, while Vietnamese populations show a CYP2C19*2 frequency of 20.50% and Malaysian populations show CYP2C19*2 and *3 frequencies of 5.70% and 6.50%, respectively (Nakhonsri et al., 2024). The frequencies of non-functional alleles CYP2C19*2 and CYP2C19*3 are notably higher than those in European populations. In this study, the frequencies of CYP2C19*2 (34.11%) and *3 (4.88%) are consistent with these Asian findings. The incidence of PMs in the Taizhou area was 15.12%, which, while close to the East Asian average (13%), is substantially higher than that observed in Europeans (2%), Africans (5%), and Native Americans (1%) (Nguyen et al., 2022). Similarly, Thai populations have been reported to show IM and PM frequencies of 42.65% and 8.53%, respectively, while Malaysian and Vietnamese populations also exhibit a substantial burden of CYP2C19 loss-of-function phenotypes (Nakhonsri et al., 2024). Studies (Koopmans et al., 2021) have demonstrated that the frequency of the *CYP2C19* PM phenotype is significantly higher in East Asian populations than in European and African populations. This marked ethnic disparity further underscores the importance of *CYP2C19* genotyping in East Asian populations. A global study on the distribution of *CYP2C19* risk phenotypes indicated differences in metabolic phenotypes among different ethnic groups (Biswas, 2021). Furthermore, an investigation involving 141,614 subjects worldwide (Zhou and Lauschke, 2022) showed that the proportion of PMs was 15%, whereas this proportion was only 3% in European populations. Direct adoption of clinical protocols derived from non-Asian populations may increase the risk of treatment failure in Chinese patients.

## Clinical implications

The most clinically significant finding of this study is that non-normal metabolizers (IMs and PMs) carrying LOF alleles account for as much as 62.85% of the Taizhou population, highlighting the necessity of genotype-guided antiplatelet therapy strategies. Given the high prevalence of LOF allele carriers in this region, it is recommended that clinicians routinely consider *CYP2C19* genetic testing when providing antiplatelet therapy for patients with ACS or those following PCI, to facilitate the transition from empirical therapy to genotype-guided precision medicine. A meta-analysis of 11 randomized controlled trials (RCTs) demonstrated that, in patients with coronary heart disease or undergoing PCI, genotype-guided antiplatelet therapy reduces the risk of major adverse cardiovascular events (MACE) without increasing the risk of bleeding compared with standard treatment (Tang et al., 2022). In recent years, *CYP2C19* testing to guide the rational clinical use of clopidogrel has been recommended by an increasing number of guidelines. The guidelines established by the Center of Excellence in Regulatory Science and Innovation in Pharmacogenetics (CERSI-PGx) (Dello Russo et al., 2026) state that pharmacogenomic testing should be conducted and integrated into existing clinical pathways to identify clinically significant *CYP2C19* polymorphisms and optimize antiplatelet therapy. The Korean Society for Laboratory Medicine has issued clinical practice guidelines for pharmacogenomic testing, identifying *CYP2C19*–clopidogrel as a gene–drug pair supported by strong clinical evidence (Rim et al., 2025). These guidelines emphasize that *CYP2C19* genotyping can assist clinicians in selecting appropriate antiplatelet agents and determining optimal dosing strategies. Furthermore, pharmacogenomic testing may complement therapeutic drug monitoring by enabling pre-treatment assessment of drug suitability and potential risks. The Dutch Pharmacogenetic Working Group (DPWG) guidelines (Beunk et al., 2024) indicate that *CYP2C19* testing is crucial before or shortly after starting clopidogrel therapy; PM patients undergoing PCI or those who have experienced a stroke or transient ischemic attack (TIA) should avoid clopidogrel. According to the Clinical Pharmacogenetics Implementation Consortium (CPIC) guidelines (Lee et al., 2022), when IM and PM patients receive standard doses of clopidogrel, active metabolite generation is reduced and platelet inhibition is lowered, thereby increasing the risk of MACE, such as stent thrombosis.

Notably, this study found that gender and age had no significant effect on the distribution of *CYP2C19* genotypes (*P* > 0.05). These findings suggest that *CYP2C19* genetic testing strategies in this region should not be restricted to specific age or gender subgroups; rather, all high-risk cardiovascular populations should be considered potential beneficiaries of genetic testing.

In addition to genetic variation, non-genetic factors may also contribute substantially to interindividual variability in clopidogrel response. Drug–drug interactions are particularly important, as certain proton pump inhibitors, especially omeprazole and esomeprazole, may inhibit *CYP2C19*-mediated bioactivation of clopidogrel. Other clinical factors, including diabetes mellitus, comorbid conditions, and medication adherence, may also influence platelet response and treatment effectiveness. Therefore, optimization of antiplatelet therapy should integrate both pharmacogenomic and non-genetic determinants (Akkaif et al., 2021; Algabbani et al., 2025).

## Strengths and limitations

Despite the significant sample size, this study has certain limitations. First, as a retrospective study, it only analyzed the frequency distribution of genotypes and phenotypes without directly correlating them with clinical endpoints, such as major adverse cardiovascular events (MACE), stent thrombosis, or bleeding outcomes. Prospective cohort studies are needed in the future to evaluate the actual clinical benefit of genotype-guided antiplatelet therapy in reducing ischemic events while also assessing potential bleeding risk. Second, this study was based on the commercially available assay routinely used in clinical practice at our institution during the study period, which primarily covered the CYP2C19*2 and *3 loci most common in East Asian populations and did not detect rare alleles such as CYP2C19*17 (the ultrarapid metabolizer allele). The CYP2C19*17 variant is associated with increased enzyme activity and may theoretically increase the risk of clopidogrel-related bleeding in elderly patients. Although the prevalence of these alleles is extremely low in East Asian populations, broader variant screening would help further improve the accuracy of phenotype prediction. Third, this study was not designed as a disease-specific case-control study. Because no matched healthy control group was included and no predefined case-control structure was available for specific cardiovascular or cerebrovascular disease subgroups, odds ratio analyses for genotype-related disease susceptibility were not feasible within the current study framework. Future studies with matched controls and disease-specific subgroup analyses are warranted to better evaluate genotype-related risk associations. Finally, in addition to *CYP2C19* genetic polymorphism, non-genetic factors may also influence clopidogrel response. In particular, drug–drug interactions, especially concomitant use of proton pump inhibitors such as omeprazole and esomeprazole, may affect *CYP2C19*-mediated clopidogrel metabolism. Because these factors were not systematically evaluated in the present study, their potential contribution to interindividual variability could not be fully assessed.

## Future perspectives

Future research should focus on prospective cohort studies integrating *CYP2C19* genotypes with detailed clinical outcomes, including major adverse cardiovascular events and bleeding complications, to further validate the clinical utility of genotype-guided antiplatelet therapy in this regional population. In addition, implementation studies are warranted to evaluate the feasibility, cost-effectiveness, and clinical workflow integration of routine *CYP2C19* testing within local cardiovascular care pathways. Given the high prevalence of loss-of-function alleles observed in this study, health economic evaluations may help determine whether preemptive pharmacogenomic screening provides long-term clinical and economic benefits. Furthermore, expanding genetic testing panels to include additional functional alleles and exploring gene–drug–environment interactions may improve the precision of phenotype prediction and optimize individualized treatment strategies. These efforts will facilitate the translation of regional pharmacogenomic evidence into standardized precision medicine practice.

In summary, this large population-based pharmacogenomic study demonstrates a high prevalence of *CYP2C19* loss-of-function alleles in patients with cardiovascular and cerebrovascular conditions from Taizhou. Given that more than 60% of individuals are non-normal metabolizers, integration of *CYP2C19* genotyping into local cardiovascular care pathways may be clinically justified. These findings provide region-specific pharmacogenomic evidence to support the integration of genotype-guided strategies into cardiovascular precision medicine practice.

## Data Availability

The datasets presented in this study can be found in online repositories. The names of the repository/repositories and accession number(s) can be found in the article/Supplementary Material.
