# Correction: Distribution of *CYP2C19* genetic polymorphisms and pharmacogenomic implications in 11,710 patients with cardiovascular and cerebrovascular conditions: a large population-based study in eastern China

**DOI:** 10.3389/fgene.2026.1925477

**Published:** 2026-07-15

**Authors:** Minfei Peng, Minmin He, Ying Chen, Xiaoli Zhu, Jun Li

**Affiliations:** 1 Department of Laboratory Medicine, Taizhou Hospital of Zhejiang Province Affiliated to Wenzhou Medical University, Linhai, China; 2 Key Laboratory of System Medicine and Precision Diagnosis and Treatment of Taizhou, Taizhou, China

**Keywords:** clopidogrel, *CYP2C19*, genetic epidemiology, genetic polymorphism, pharmacogenomics

The ethics statement was erroneously given as “The studies involving humans were approved by Ethics Committee Approval Letter of Biomedical Research Involving Humans. The studies were conducted in accordance with the local legislation and institutional requirements. The participants provided their written informed consent to participate in this study.” The correct ethics statement is “The studies involving humans were approved by the Medical Ethics Committee of Taizhou Hospital of Zhejiang Province (Approval No. K20260129). The study was conducted in accordance with local legislation and institutional requirements.”

The original version of this article has been updated.

